# Intrinsic Circuits in the Lateral Central Amygdala

**DOI:** 10.1523/ENEURO.0367-16.2017

**Published:** 2017-03-24

**Authors:** Sarah Hunt, Yajie Sun, Hakan Kucukdereli, Rüdiger Klein, Pankaj Sah

**Affiliations:** 1 Queensland Brain Institute, The University of Queensland, Brisbane 4072, Queensland, Australia; 2 Max Planck Institute of Neurobiology, 82152 Martinsreid, Germany

**Keywords:** anxiety, emotion, fear, learning

## Abstract

Network activity in the lateral central amygdala (CeL) plays a crucial role in fear learning and emotional processing. However, the local circuits of the CeL are not fully understood and have only recently begun to be explored in detail. Here, we characterized the intrinsic circuits in the CeL using paired whole-call patch-clamp recordings, immunohistochemistry, and optogenetics in C57BL/6J wild-type and somatostatin-cre (SOM-Cre) mice. Our results revealed that throughout the rostrocaudal extent of the CeL, neurons form inhibitory connections at a rate of ∼29% with an average amplitude of 20 ± 3 pA (at −40 mV). Inhibitory input from a single neuron is sufficient to halt firing in the postsynaptic neuron. *Post hoc* immunostaining for protein kinase Cδ (PKCδ) in wild-type mice and paired recordings in SOM-Cre mice demonstrated that the most common local connections were PKCδ(−) → PKCδ(−) and SOM(+) → SOM(+). Finally, by optogenetically activating either SOM(+) or SOM(−) neurons, we found that almost all neurons in the CeL were innervated by these neuronal populations and that connections between like neurons were stronger than those between different neuronal types. These findings reveal a complex network of connections within the CeL and provide the foundations for future behavior-specific circuit analysis of this complex network.

## Significance Statement

Local inhibition in the lateral central amygdala (CeL) plays a crucial role in the processing of emotions, yet a complete understanding of these connections is still in its infancy. In this study, we show that CeL neurons are highly interconnected and that inhibition from a single neuron is sufficient to silence the postsynaptic neuron. Focusing on two well known CeL neuronal subtypes, protein kinase Cδ (PKCδ)-expressing and somatostatin (SOM)-expressing neurons, we show that the most common local connections are PKCδ(−) → PKCδ(−) and SOM(+) → SOM(+). Optogenetic activation of either the SOM(+) or SOM(−) neuronal populations revealed that inhibition was larger between like neurons. These findings show that within the CeL there is a complex network and provide the foundations for future behavior-specific circuit studies.

## Introduction

The amygdala has long been known to play a crucial role in processing innate emotions, particularly fear (Klüver and Bucy, 1939; Weiskrantz, 1956; Sah et al., 2003). In Pavlovian fear conditioning, an associative learning paradigm widely used to study amygdala function, subjects learn to associate a neutral sensory stimulus [the conditioned stimulus (CS)], with an aversive one (the unconditioned stimulus; LeDoux, 2000). Following learning, the previously neutral CS now evokes a defensive response (i.e., freezing of movement or flight; Gross and Canteras, 2012). A converging body of evidence has established the amygdala as a central player in fear conditioning where the basolateral amygdala (BLA) and the central amygdala (CeA) are the key sites involved in the acquisition and expression of fear (LeDoux, 2000; Sah et al., 2003; Duvarci and Pare, 2014). The BLA has been extensively studied with respect to its cell types, intrinsic circuits, and extrinsic connections (LeDoux, 2000; Sah et al., 2003; Duvarci and Pare, 2014), while the CeA has received considerably less attention and the intrinsic circuits within this nucleus are less well understood.

The CeA is a GABAergic nucleus (McDonald and Augustine, 1993; Sun and Cassell, 1993) that is anatomically divided into the lateral sector of the CeA (CeL) and the medial sector of the CeA (CeM), with substantial unidirectional connections between the CeL and the CeM (McDonald, 1982; Grove, 1988; Jolkkonen and Pitkänen, 1998). Neurons in both regions also make extensive local connections (McDonald, 1982; Sun and Cassell, 1993; Jolkkonen and Pitkänen, 1998), with local glutamate excitation of CeL neurons evoking IPSCs in neighboring neurons (Lopez de Armentia and Sah, 2004). Recent studies have divided CeL neurons into distinct populations based on the expression of immunohistochemical markers, electrophysiological properties, and synaptic connections (Ciocchi et al., 2010; Haubensak et al., 2010; Li et al., 2013). Of these, one population expresses protein kinase Cδ [PKCδ(+)], and these neurons are predominantly described as late-firing (LF) neurons, exhibiting a substantial delay to action potential (AP) initiation in response to depolarizing somatic current injections. Following fear conditioning, these neurons respond to the CS with a reduction in activity and have therefore been called CeL_OFF_ cells (Ciocchi et al., 2010; Haubensak et al., 2010). A second population of CeL neurons, which is largely separate from the PKCδ(+) population, expresses somatostatin [somatostatin-positive (SOM+); Li et al., 2013]. These neurons receive direct synaptic input from the lateral amygdala, which is potentiated following auditory fear conditioning (Li et al., 2013). Electrophysiologically, PKCδ(−) neurons which are predominantly SOM(+), have been described as either LF or regular spiking (RS). Following fear conditioning, PKCδ(−) neurons respond to the CS with an increase in activity, and have therefore been called CeL_ON_ neurons (Ciocchi et al., 2010; Haubensak et al., 2010), which likely also correspond to SOM(+) neurons (Yu et al., 2016). PKCδ(−) neurons inhibit PKCδ(+) neurons, which in turn project to the CeM (Haubensak et al., 2010).

This organization has led to one model in which fear expression is mediated by CS-related information driving PKCδ(−) neurons, presumably SOM(+) neurons, in the CeL via excitatory input from the BLA and thalamus. These neurons in turn inhibit PKCδ(+) neurons, resulting in disinhibition of the CeM and the expression of fear (Ciocchi et al., 2010; Haubensak et al., 2010). However, some SOM(+) neurons in the CeL also project to the periaqueductal gray (PAG; Penzo et al., 2014), and CS-driven activity of these neurons also contributes to fear expression (Tovote et al., 2016). Moreover, recent studies have reported that neurons in the CeL are also involved in feeding (Cai et al., 2014), and pain (Han et al., 2015). Neurons engaged during feeding and pain responses are also part of the PKCδ and SOM population, indicating that the intrinsic circuitry of the CeL is complex, and the strength, identity, and physiologic role of individual local connections are not fully understood. In this study, we provide a detailed investigation of local circuits in the CeL.

## Materials and Methods

### Animals

All studies were approved by the University of Queensland Animal Ethics Committee, and experiments were conducted in accordance with the Australian Code of Practice for the Care and Use of Animals for Scientific purposes. Adult (6–15 weeks old) male wild-type C57BL/6J mice were used for electrophysiology experiments. Where stated, we also used both male and female mice (8–12 weeks old) from a somatostatin-IRES-cre mouse line (SOM-Cre; C57BL/6J background; Sst^tm2.1(cre)Zjh^) that was acquired from The Jackson Laboratory. These mice express cre recombinase under the SOM promoter, thereby allowing selective targeting of SOM(+) neurons using cre-dependent viral constructs (described below). Mice were genotyped by the Australian Equine Genetics Research Center.

### Brain slice preparation

Mice were anesthetized using isoflurane and decapitated, after which brains were quickly removed while submerged in an oxygenated ice-cold *N*-methyl-d-glucamine-based (NMDG) solution (NMDG 93 mm, KCl 2.5 mm, NaH_2_PO_4_ 1.2 mm, NaHCO_3_ 30 mm, HEPES 20 mm, glucose 25 mm, sodium ascorbate 5 mm, thiourea 2 mm, sodium pyruvate 3 mm, MgSO_4_ 10 mm, and CaCl_2_ 0.5 mm, pH 7.2, 290–300 mOsm). This NMDG-based solution is particularly suited for dissections of adult mice (Zhao et al., 2011). Coronal brain slices (300 μm thick) were then prepared using a vibratome (catalog #VT1000S, Leica) and placed to recover in oxygenated artificial CSF (aCSF; NaCl 118 mm, NaHCO_3_ 25 mm, glucose 10 mm, KCl 2.5 mm, NaHPO_4_ 1.2 mm, MgCl_2_ 1.3 mm, and CaCl_2_ 2.5 mm, pH 7.2, 290–300 mOsm) for 30 min at 34°C, and then at room temperature until required.

### Electrophysiological recordings

Slices were visualized on an upright microscope (model BX51WI, Olympus), and whole-cell patch-clamp recordings were made using a Multiclamp 700B (Molecular Devices). The CeL was easily distinguishable *in vitro* based on the fire bundles that surround and clearly delineate this area (see Fig. 4*A*). These landmarks are readily visible under the microscope and ensured that cells chosen for recordings were situated within the CeL. In addition, for electrophysiological recordings, cells in the CeL are typically smaller than those in the BLA, and their cell density is higher than both the BLA and CeM. Data were filtered at 4 kHz and sampled at 20 kHz using an ITC-18 (Instrutech). Data were acquired and analyzed using AxoGraphX software (AxoGraph). Brain slices were continuously perfused with oxygenated aCSF (34°C; 3–4 mL/min), and recording electrodes (4–6 MΩ; glass capillaries, Harvard Apparatus; PC-10 Electrode Puller, Narishige) were filled with a KMeSO_4_-based internal solution (KMeSO_4_ 135 mm, NaCl 8 mm, HEPES 10 mm, MgATP 2 mm, GTP 0.3 mm, phosphocreatine 7 mm, EGTA 0.2 mm, and biocytin 0.2%, pH 7.2 with KOH, osmolarity 295 mOsm/kg) unless otherwise stated, in which case a CsMeSO_4_-based internal solution was used (CsMeSO_4_ 135 mm, NaCl 8 mm, HEPES 10 mm, MgATP 2 mm, GTP 0.3 mm, phosphocreatine 7 mm, and spermine 0.1 mm, pH 7.2 with CsOH, osmolarity 300 mOsm/kg). In some experiments GABA (10 mm) was added to the KMeSO_4_-based internal solution to avoid any run down of responses due to wash out during whole-cell recordings (Apostolides and Trussell, 2013), although no difference in response was observed when using GABA internal solutions. No corrections were made for junction potentials. The pairs of neurons chosen for recordings were located within 50–100 µm of each other in the coronal plane and 10–40 µm in the rostrocaudal plane. To probe for connections during paired recordings, one cell was held in current-clamp mode and injected with a 5 ms, 600–700 pA current pulse to evoke an AP. Meanwhile, the second (postsynaptic) neuron was held in voltage-clamp mode at −40 mV, well away from the chloride reversal potential (approximately −73 mV), given that neurons in the CeL are known to be GABAergic, forming inhibitory synapses (Sun and Cassell; Lopez de Armentia and Sah, 2004; Haubensak et al., 2010; Li et al., 2013). This protocol was repeated for at least 20 (but not >50) episodes, and sweeps were averaged for analysis. The same was then done in the opposite direction. Only connections with an amplitude of >5 pA were considered to be connected. Finally, in pharmacology experiments, bicuculline (10 μm; Sigma-Aldrich) or CNQX (10 μm; Tocris Bioscience) were bath applied to the slice.

### Firing properties

APs were evoked using current injections applied in increments of 20 pA from −60 to 240 pA. AP threshold, amplitude, delay, half-width, rise time, and spike accommodation were analyzed off-line (described below). Spike accommodation was measured as the difference in AP frequency over at least eight APs at twice threshold. Although the two main firing types we observed ultimately had significantly different AP onsets, we used the absence or presence of spike accommodation to classify these firing types, as AP onset varied with small changes in holding membrane potential.

### Data analysis

#### Electrophysiological properties

Resting membrane potential (*R*_m_) was recorded on-line immediately after break-in, whereas input resistance (*R_i_*) was measured off-line as *R_i_* = *dVm*/*l*, where *dVm* is the change in membrane potential in response to a −20 pA (800 ms) current injection (*l*). For connections, decay was measured by fitting the average IPSC by a sum of two exponentials (simplex sum of squared errors) to calculate a weighted time constant: $\tau_{w}=\frac{\left(t_{1}\cdot a_{1}+t_{2}\cdot a_{2}\right)}{{(a}_{1}+a_{2})}$. Onset delay was calculated as the difference between the time of the presynaptic AP peak and the time of IPSC onset (time at 5% of peak). For firing properties, AP threshold was measured as the membrane potential at the start of the fast-rising phase. AP amplitude was measured from the threshold to peak, and delay was measured as the duration from the start of the current injection to the start of the fast-rising phase of the first AP.

#### Statistical tests

Datasets were tested for normality using the Shapiro–Wilks test. In the cases where a subset of the population was tested (e.g., drug application), we based our choice of statistical test on whether or not the overall dataset was normally distributed. We used parametric tests (*t* tests) when the data followed a normal distribution, whereas nonparametric tests (Wilcoxon and Mann–Whitney tests) were used for datasets that were too small to reliably test for or did not follow a normal distribution. Two-tailed tests were used unless otherwise stated, and differences were considered to be significant at *p* < 0.05.

### Immunohistochemistry

#### Labeling for immunohistochemical characterization

For characterization of CeA neurons, mice were anesthetized by intraperitoneal injection of pentobarbitone sodium (3250 mg/kg; Virbac) and transcardially perfused with 40 ml of a 1% sodium nitrite solution (phosphate buffer, 0.1 m), followed by 40 ml of 4% paraformaldehyde (PFA; in 0.1 m phosphate buffer). Brains were then removed and left in 4% PFA at room temperature overnight and washed (3 × 15 min, PBS 0.1 m) before sectioning (50–60 μm sections). Brains were placed in 30% sucrose for 48 h and sectioned using a sliding microtome (model SM200R, Leica). Coronal subsections (50 µm) were then stained for PKCδ using a mouse anti-PKCδ antibody (1:500; BD Biosciences), for SOM using a rabbit anti-SOM antibody (1:1000; Millipore Bioscience Research Reagents/Millipore), and for NeuN using a chicken anti-NeuN antibody (1:1000; Millipore; 72 h at room temperature). In the case of virus-injected animals, fluorescence was amplified using either a rabbit anti-red fluorescent protein antibody (1:1000; Abcam) or chicken anti-green fluorescent protein (1:1000; Life Technologies). Sections were then washed and incubated with mouse-fluorophore 647 (for PKCδ; 1:2000; Invitrogen), rabbit-fluorophore 488 (for SOM; 1:2000; Invitrogen), rabbit-fluorophore 568, or chicken-fluorophore 488 (for fluorescence-enhanced sections; 1:2000; Invitrogen). Brain sections used for counts were immunolabeled for NeuN to allow reliable identification of mature neurons, and only NeuN(+) neurons were counted. Cell counts were made in both the right and left hemispheres, but, because these were not significantly different, the data were pooled for each bregma location.

#### *Post hoc* labeling of recorded neurons

Alexa Fluor 568 (1 ng/ml internal solution) was added to the internal recording solution, and images of dendritic morphology were taken during recordings to correctly identify the presynaptic and postsynaptic cells after recovery of recorded neurons. Following electrophysiological recordings, slices were fixed in 4% PFA (in 0.1 m phosphate buffer) for either 1 h at room temperature or overnight at 4°C, and then washed for 3 × 15 min in 0.1 m PBS. Slices were then placed in blocking solution (1% BSA, 0.05% saponin, and 0.05% sodium azide) for 1–2 h at room temperature before incubation with an Alexa Fluor 555-bound streptavidin (overnight at room temperature; 1:2000 in blocking solution; Life Technologies). Slices were then washed (3 × 15 min, 0.1 m PBS), mounted (DABCO), and imaged using either an upright fluorescent microscope (5× and 20×; Zen Software, Zeiss) or spinning disk confocal microscope (20× and 40× water-immersion objective, model #CSU-W1, Yokogawa; Slidebook software). All images were analyzed using FIJI (ImageJ). For protein PKCδ staining, slices were subsequently embedded in 4% agarose and subsectioned (50 μm sections; VT1000S vibratome, Leica) before being incubated with the PKCδ mouse-antibody (72 h at room temperature; 1:500; BD Biosciences). Sections were then washed and incubated with mouse-fluorophore 647 (1:2000; Invitrogen), and the nuclei of the cells stained with DAPI, before being mounted and imaged as described above. Although PKCδ clearly labeled somas, the somatostatin antibody did not deliver reliable *post hoc* staining, as a result of which we focused on PKCδ for postrecording labeling experiments.

#### Morphology

Biocytin-recovered neurons that were used for morphologic reconstruction were imaged using a spinning disk confocal microscope (40 × 1.2 numerical aperture water-immersion objective, 0.156 × 0.156 × 0.33 μm^3^/pixel resolution; model #CSU-W1, Yokogawa; Slidebook software). Neurons were manually traced using Neurolucida (MBF Bioscience) and analyzed using Neurolucida Explorer. For spine counts, dendrites were reimaged using a 63 × 1.4 numerical aperture oil-objective (0.099 × 0.099 × 0.15 μm^3^/pixel resolution) and underwent deconvolution. Spines were counted automatically and manually verified (Neurolucida 360, MBF Bioscience; including the *z*-plane) over 60 μm of secondary dendrites. Three segments (each from a different secondary dendrite) were counted and averaged for each cell.

### Viral injections and optical stimulation

Mice (21–28 d old) were anesthetized (100 mg/kg ketamine, 10 mg/kg xylazil in saline) and placed in a stereotaxic frame. Bilateral injections were made into CeL using the following coordinates (Paxinos and Watson, 2001): −1.6 mm (anteroposterior); ± 2.8 mm (mediolateral); and −4.8 mm (dorsoventral from skull).

A small hole was drilled in the skull, and virus was injected using a glass needle (pressure injection Picospritzer; 10–20 ms, 10–30 psi). Animals were injected stereotaxically with an AAV (adeno-associated virus; 0.1–0.3 μl, 0.1 μl/min Vector Core) containing one of the following constructs: AAV2/5- EF1α-DIO-tdTomato (titer: 1.0 × 1011); AAV2/5- EF1α.dflox.hChR2(H134R)-mCherry (titer: 1.31 × 1013); or AAV2/5-EF1α-DIO-Fwd.hChR2(H134R)-EYFP (enhanced yellow fluorescent protein; titer: 1.0 × 1011).

Animals were quarantined for 48 h then allowed to recover for at least 4 weeks postinjection. Brain slices were prepared as described above for electrophysiological experiments, and cells were only recorded well within the spread of the virus to ensure that nonfluorescent neurons were indeed SOM(−) rather than simply not infected. To verify the expression of channelrhodopsin (ChR2) and to activate ChR2 in infected cells, an LED system (470 nm, 1.4 mW; pE-2 LED System, CoolLED) attached to the microscope (via the rear C-mount port) was used. A prolonged light pulse (100 ms) was used to verify that cells expressed functional ChR2. In the case of AAV2/5- EF1α.dflox.hChR2(H134R)-mCherry experiments, for example, neurons were considered SOM(+) if they were both fluorescent and displayed a prolonged depolarization in response to prolonged light stimulation (470 nm, 100 ms), whereas a SOM(−) neuron was not fluorescent and showed no excitation to the light pulse. A light pulse of 2 ms (*n* = 57 neurons) or 1 ms (*n* = 10 neurons) was used to evoke responses in the CeL.

## Results

### Characterization of neurons in the central lateral amygdala

#### Immunohistochemical characterization

**Figure 1.**
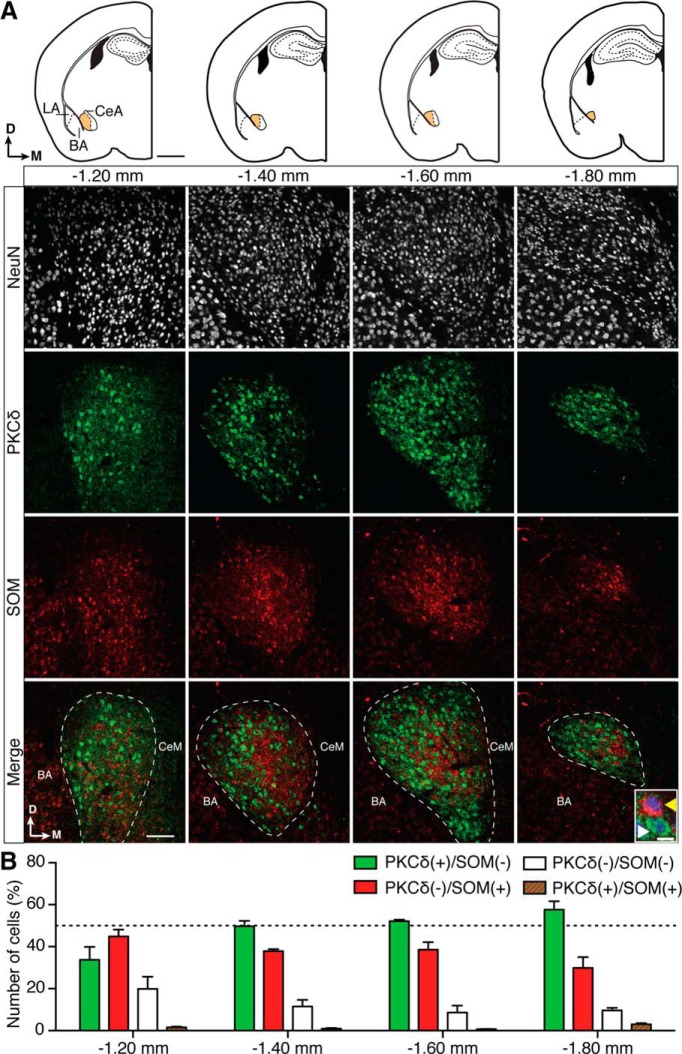
PKCδ and SOM label distinct populations of neurons in the CeL of wild-type C57BL/6J mice. ***A***, Top, Diagrams of coronal CeL slices of C57BL/6J mouse at −1.20, −1.40, −1.60, and −1.80 mm from bregma. LA, Lateral amygdala; BA, basal amygdala; CeA, central amygdala, which is divided into the CeL (orange) and the central medial amygdala (CeM, in white). Arrows show dorsal and medial orientation. Scale bar, 1 mm. Bottom panels show closeups of the CeL in 50 μm sections that were stained for NeuN (to stain somas of neurons, white fluorescence), PKCδ (green fluorescence), and SOM (red fluorescence). Scale bar in bottom left square, 100 μm. For clarity, the merged panels represent the merging of PKCδ and SOM only. The CeL is outlined in the bottom panel, and this outline was defined both by landmarks visible in bright field (data not shown), and the presence of PKCδ(+) somas. PKCδ(+) fibers can typically be seen in the CeM. The locations of both the BA and the CeM are also labeled in the merged panels, and note that by 1.80 mm the CeM is no longer present. The inset in the lower right corner of the far right merged panel shows a closeup of the most common cells types: PKCδ(+)/SOM(−) (white arrowhead) and SOM(+)/PKCδ(−) neurons (yellow arrowhead; scale bar, 10 μm; PKCδ green fluorescence, SOM red fluorescence, NeuN blue fluorescence). ***B***, Only NeuN(+) neurons were counted to ensure that only mature neuronal cells were taken into account. Of these, 48 ± 5% were PKCδ(+)/SOM(−) (mean *n* = 83 ± 19 neurons/1.0 × 10^−3^ mm^3^), and 38 ± 3% were SOM(+)/PKCδ(−) (mean *n* = 66 ± 14 neurons/1.0 × 10^−3^ mm^3^). These two populations were largely distinct as only 1 ± 0.5% of neurons were PKCδ(+)/SOM(+) (mean *n* = 2 ± 0.3 neurons/1.0 × 10^−3^ mm^3^), and 12 ± 2% NeuN(+) cells were PKCδ(−)/SOM(−) (mean *n* = 20 ± 4 neurons/1.0 × 10^−3^ mm^3^). The dotted line on the graph indicates 50% and bregma-specific percentages were as follows: PKCδ(+)/SOM(−), 34 ± 6% (−1.20 mm), 50 ± 2% (−1.40 mm), 52 ± 1% (−1.60 mm), and 57 ± 4% (−1.80 mm). PKCδ(−)/SOM(+), 45 ± 3% (−1.20 mm), 38 ± 1% (−1.40 mm), 39 ± 3% (−1.60 mm), and 30 ± 5% (−1.80 mm); PKCδ(−)/SOM(−), 20 ± 6% (−1.20 mm), 11 ± 3% (−1.40 mm), 8 ± 3% (−1.60 mm), and 10 ± 1% (−1.80 mm); and PKCδ(+)/SOM(+), 1 ± 0.3% (−1.20 mm), 1 ± 0.2% (−1.40 mm), 1 ± 0.05% (−1.60 mm), and 3 ± 0.5% (−1.80 mm).

Neurons in the CeL have been separated based on the expression of a range of neuropeptides and markers that include PKCδ, SOM, corticotropin-releasing factor, oxytocin receptors, enkephalin, and others (Cassell and Gray, 1989; Haubensak et al., 2010). Of these, the two most highly expressed and clearly distinct neuropeptides are PKCδ and SOM (Haubensak et al., 2010; Li et al., 2013). Immunostaining of brain sections from four locations posterior to bregma (−1.20, −1.40, −1.60, and −1.80 mm; ±0.05 mm; Fig. 1*A*, top diagrams) shows that PKCδ labeling within the amygdala was specific to the CeL, whereas SOM expression was also present outside the central amygdala. In the CeL, 48 ± 5% of neurons expressed PKCδ, and 38 ± 3% SOM (Fig. 1*A*), with the two populations largely nonoverlapping, and dual-labeled [PKCδ(+)/SOM(+)] neurons accounting for only 1.5 ± 0.5% of neurons. The remaining neurons (13 ± 2%) were negative for both markers. It was notable that whereas the proportions of PKCδ(+)/SOM(−) and PKCδ(−)/SOM(+) neurons were similar between bregma −1.40 and −1.60 mm, the difference between the total numbers of the two cell types changed at bregma −1.20 and −1.80 mm, the rostral and caudal limits of the CeL (Fig. 1*B*).

#### Electrophysiological properties

**Figure 2.**
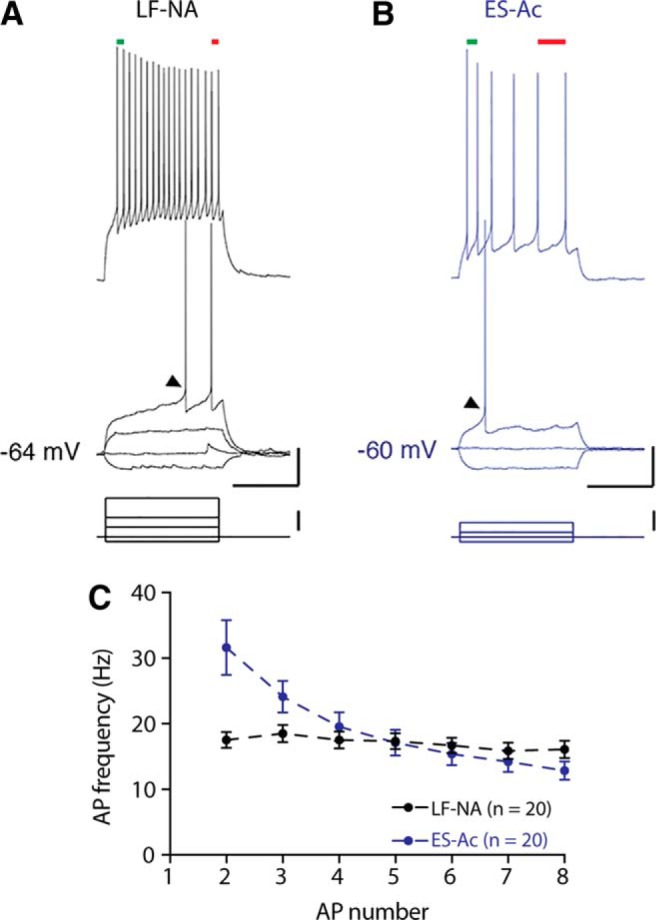
Firing types of neurons in the central lateral amygdala are as follows: late-firing nonaccommodating and early-spiking accommodating. ***A***, ***B***, Example traces of the two main firing types recorded in the CeL: LF-NA (***A***) and ES-Ac (***B***) with example traces of current injections below. Calibration: 20 mV, 500 ms, 80 pA. The top two current injections shown are at threshold and twice threshold (2T). On average, LF-NA neurons displayed significantly longer onset to firing of the first AP (onset indicated by black arrowheads) when compared with ES-Ac neurons (LF-NA, 330 ± 25 ms, *n* = 80 neurons; ES-Ac, 209 ± 23, *n* = 59 neurons; *p* < 0.001, Mann–Whitney test) and little to no accommodation at 2T. To demonstrate accommodation, early (green lines) and late (red lines) interspike intervals are indicated. ***C***, Whereas AP frequency over eight action potentials remained consistent for LF-NA neurons (*n* = 20; AP_1-2_ frequency, 17 ± 1 Hz; AP_7-8_ frequency, 16 ± 1 Hz; *p* = 0.6, Wilcoxon matched-pairs test), ES-Ac AP frequency gradually decreased (AP_1-2_ frequency, 32 ± 4 Hz; AP_7-8_ frequency, 13 ± 1 Hz; *p* < 0.001, Wilcoxon matched-pairs test).

Based on their response to somatic current injections, three general types of CeL neurons have previously been described, with the two major types being LF neurons, which show a significant delay before onset of the first AP (∼100–200 ms), and early-spiking (ES) neurons (also described as regular-spiking; AP onset, ∼50 ms). A third, smaller population of low-threshold bursting neurons has also been described (Dumont et al., 2002; Lopez de Armentia and Sah, 2004; Haubensak et al., 2010; Li et al., 2013; Hou et al., 2016). We characterized the firing properties of 151 CeL neurons. However, while classifying neurons we found that AP onset varied with changes in holding potential, whereas the presence of spike frequency accommodation was more reliable. Using this measure, neurons were classified either as nonaccommodating (NA), where AP frequency remained relatively consistent (∼17 Hz), or accommodating (Ac), where there was clear spike frequency adaptation (AP_1-2_, ∼32 Hz; AP_7-8_, 13 Hz; *p* < 0.001 Wilcoxon matched-pairs test; Fig. 2*C*). The large majority of our neurons were nonaccommodating (*n* = 80 neurons; Fig. 2*A*) or accommodating (*n* = 59 neurons; Fig. 2*B*). Nonaccommodating neurons also had a significantly longer mean onset compared with that of accommodating neurons (Table 1), and these neurons generally corresponded to the LF and ES types (Haubensak et al., 2010; Amano et al., 2012). Thus, for consistency we have termed these LF-NA and ES-Ac neurons. Apart from resting membrane potential, which was significantly more depolarized in ES-Ac neurons, other membrane properties such as input resistance, threshold potential, AP amplitude, rise time, and half-width did not differ significantly between LF-NA and ES-Ac neurons (Table 1).

**Table 1:** Membrane properties of neurons in the central lateral amygdala

Firing type	Nonaccommodating (*n* = 80)	Accommodating (*n* = 59)	Stuttering (*n* = 12)
Incidence	53%	39%	8%
Input resistance (mΩ)	416 ± 17	419 ± 28	387 ± 64
Resting potential (mV)	-64 ± 1	-59 ± 1^a^	-62 ± 2
Threshold (mV)	-33 ± 0.5	-34 ± 0.5	-34 ± 1.8
Onset (ms) at T	330 ± 25	209 ± 23^b^	122 ± 54
Onset (ms) at 2T	77 ± 5	59 ± 7^c^	28 ± 19
Amplitude (mV)	66 ± 1	69 ± 1	53 ± 4^d,e^
Rise time (ms)	0.4 ± 0.02	0.4 ± 0.02	0.2 ± 0.02^f,g^
Half-width (ms)	1.2 ± 0.03	1.1 ± 0.03	0.6 ± 0.04^f,h^

Values are the mean ± SEM. Low-threshold bursting neuron properties are not represented in this table since *n* = 1 for this firing type. T, Threshold; 2T, twice threshold.

a *p* < 0.001 vs NA (two-tailed *t* test).

b *p* < 0.001 vs NA (Mann–Whitney test).

c *p* < 0.01 vs NA (Mann–Whitney test)

d *p* < 0.001 vs NA (two-tailed *t* test).

e *p* < 0.0001 vs Ac (two-tailed *t* test).

f *p* < 0.0001 vs NA (Mann–Whitney test).

g *p* < 0.001 vs Ac (Mann–Whitney test).

h *p* < 0.0001 vs Ac (Mann–Whitney test).

**Figure 3:**
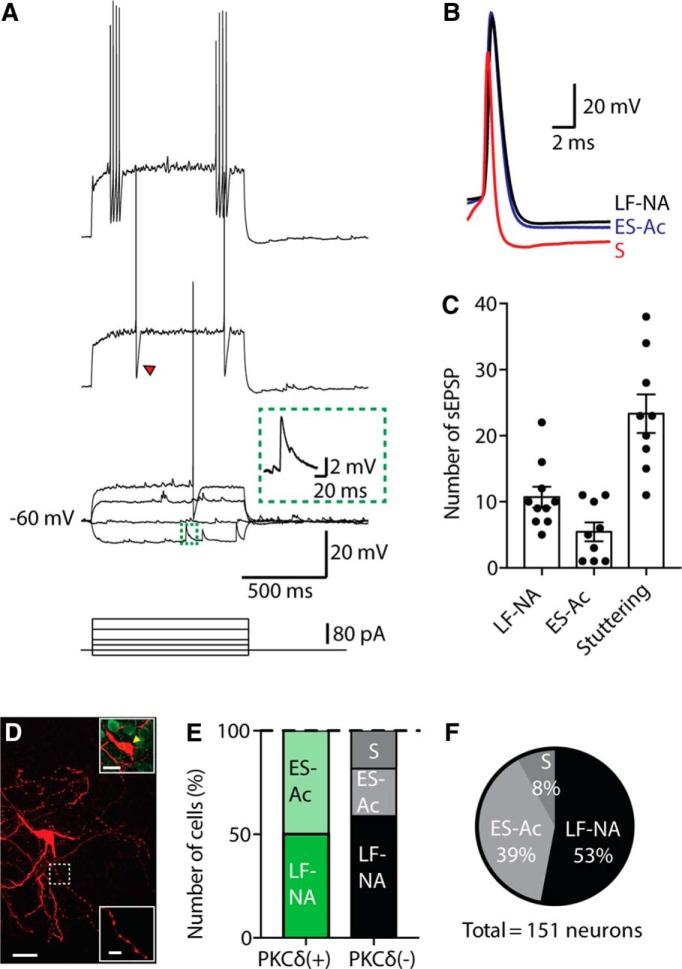
, Stuttering neurons in the CeL. ***A***, Example trace of firing of a stuttering (S) neuron at threshold, and twice and three times threshold. In addition to its fast AP kinetics (Table 1) and distinct firing pattern, large fast afterhyperpolarizations (as indicated by the red arrowhead) are also typical of this firing type. Inset shows a closeup of a spontaneous EPSP (sEPSP) in green. ***B***, Overlay of the first AP of a stuttering (red), LF-NA (black), and ES-Ac (blue) neurons. The AP rise time and half-width of S neurons were significantly faster than those of LF-NA and ES-Ac neurons (Table 1). ***C***, sEPSPs in S neurons were significantly more numerous than in LF-NA and ES-Ac neurons during the hyperpolarizing steps of this protocol. Numbers shown are the total counted over the −60, −40, and −20 pA current injections (***B***; S vs LF-NA: *p* = 0.001, unpaired *t* test; S vs ES-Ac: *p* < 0.0001, unpaired *t* test). ***D***, Example biocytin recovery of an S neuron, which was PKCδ(−) (top inset, yellow arrowhead indicates the soma of the S neuron). Scale bars: 20 µm; top inset, 10 µm; bottom inset, 5 µm. This neuron displayed an extensive axon with inset showing a closeup of the axon in the dotted white square. ***E***, Percentage of firing types for recovered neurons that were PKCδ(+) (*n* = 8) or PKCδ(−) (*n* = 17). ***F***, Shows total percentage of each firing type.

In the remaining 12 neurons (8%; Fig. 3), we found a distinct stuttering firing type that resembled that of some interneurons in the BLA (Woodruff and Sah, 2007; Sosulina et al., 2010; Spampanato et al., 2011). These neurons were easily distinguishable due to their distinctive firing pattern, with bursts of high-frequency APs (∼60 Hz; Fig. 3*A*). Moreover, these neurons had significantly briefer APs with a half-width of 0.6 ± 0.04 ms compared with 1.1 ± 0.03 ms in ES-Ac neurons and 1.2 ± 0.03 ms in LF-NA (Table 1; Fig. 3*B*). Stuttering neurons also displayed a higher frequency of spontaneous synaptic events compared with LF-NA and ES-Ac neurons (Fig. 3*C*). For stuttering neurons, we were unable to recover the entire cell; however, dendrites were filled, and visible, and showed that, unlike LF-NA and Es-Ac neuron, stuttering neurons were aspiny.

Twenty-five recorded neurons were successfully recovered with biocytin and labeled for PKCδ. Of these, PKCδ(+) neurons (*n* = 8) were either LF-NA or ES-Ac at equal incidence (50%), whereas PKCδ(−) neurons (*n* = 17) were more likely to be LF-NA (∼59%) than ES-Ac (∼23%). As previously described using Golgi methods (McDonald, 1982; Cassell and Gray, 1989), the majority of CeL neurons resembled medium-spiny neurons (see Fig. 5). Stuttering neuron somas that were successfully recovered and stained (*n* = 3) were all PKCδ(−) (Fig. 3*D*,*E*). These results show that PKCδ-expressing (48%), and SOM-expressing (38%) neurons are the major cell types in the CeL, with very few neurons expressing both markers (1.5%). These neurons have one of two firing properties, LF-NA or ES-Ac. We also identified a previously unrecognized population of stuttering neurons (8%) that express neither PKCδ or SOM (see below).

### Local inhibitory connections

**Figure 4.**
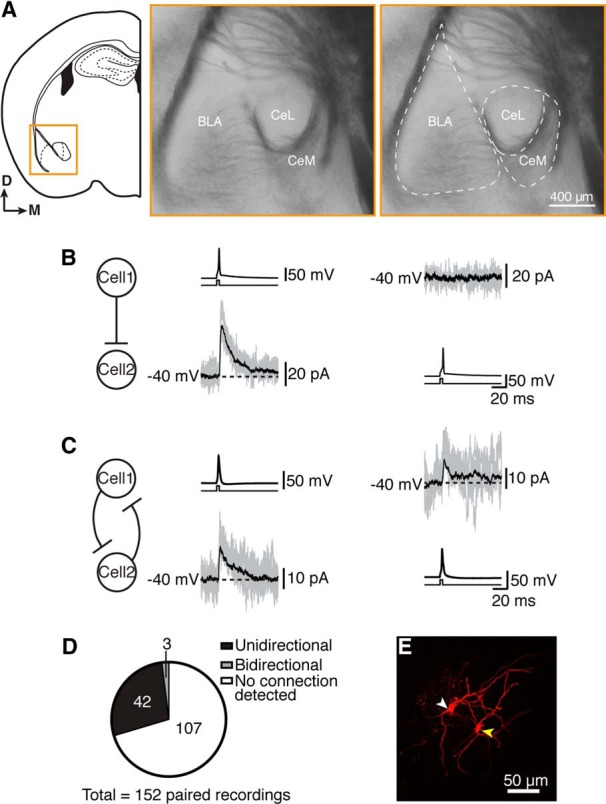
Neurons in the central lateral amygdala form local connections. ***A***, Paired recordings were performed in the CeL, the location of which is shown in a diagram of a coronal slice (left). Middle, A bright-field image (300 µm slice) of the area within the orange rectangle: the border of the CeL is clearly defined by visible fiber bundles, and the right panel shows the approximate outline of the three main amygdala regions: BLA, CeL, and CeM. In reality, the CeL extends slightly more ventrally than outlined here; however, we aimed to keep recordings within the outlined area to ensure that we did not mistakenly record from CeM neurons. ***B***, ***C***, Example traces of IPSCs, which were on average 20 ± 3 pA, from a unidirectional connection (***B***) and a bidirectional connection (***C***). In each case, “cell 1” was current clamped and given a short current injection (5 ms, 600–700 pA, illustrated in black directly under each current trace) to elicit one AP, while “cell 2” was voltage clamped at −40 mV. The protocol was then repeated in the opposite direction: from cell 2 to cell 1. Example average traces (black) and representative traces from single episodes (gray) are shown. ***D***, Approximately 29% of paired recordings (*n* = 45 of 152) were connected, with the large majority of connected pairs being unidirectional connections (42 of 45) and the remainder being bidirectional connections. ***E***, Biocytin recovery of the connected recorded pair in ***B***, where a yellow arrowhead indicates the presynaptic cell and a white arrowhead indicates the postsynaptic cell.

**Figure 5.**
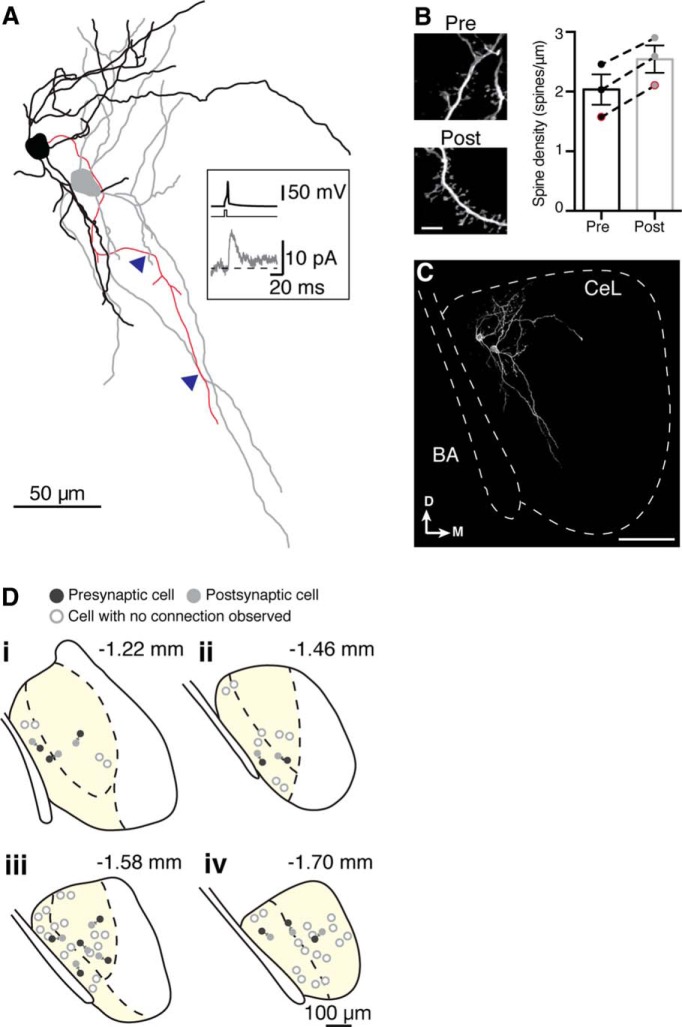
Morphology and anatomic location of local connections within the central lateral amygdala. ***A***, Example morphologic reconstruction (spines not depicted) of a connected pair with the presynaptic neuron in black and the postsynaptic neuron in gray. Blue arrowheads indicate where the presynaptic axon (red) crossed over a postsynaptic dendrite in the same *z*-plane, representing putative synapse locations. Inset shows average traces of this connection, with the presynaptic trace in black and the postsynaptic trace in gray (postsynaptic cell voltage clamped at −40 mV). ***B***, Recovered neurons typically had a medium spiny morphology; spine counts of recovered connected neurons showed that the postsynaptic neuron was not significantly more spiny than its presynaptic neuron. Example images show closeups of secondary dendrites from a presynaptic (“Pre”) neuron and corresponding postsynaptic (“Post”) neuron from the pair shown in ***A***. Scale bar, 5 µm. Bar graph shows mean spine densities (number of spines per micrometer) for presynaptic and postsynaptic neurons, with connected neurons joined by a dotted line (*n* = 3 connected pairs). Data points with red borders correspond to the Pre and Post closeups depicted in ***B***. ***C***, Image of biocytin recovery of the connected pair of neurons shown in ***A*** to show the location within the CeL. BA, Basal amygdala; D, dorsal; M, medial. Scale bar, 100 µm. ***D***, Locations within the CeL (yellow; central medial amygdala is in white) of 35 recorded pairs that could be reliably located at different rostrocaudal locations (−1.22 to −1.70 mm from bregma; ***Di–Div***). Presynaptic cells are represented by black circles, and postsynaptic cells are represented by solid gray circles. White circles indicate pairs where a connection was not detected.

To determine the nature of local connections between neurons in the CeL, paired whole-cell recordings were made in acute coronal slices of wild-type mice (Fig. 4*A*). A total of 152 pairs were tested, of which 45 (29%) were connected. This was a monosynaptic connection with an onset latency of 0.85 ± 0.06 ms after the AP peak and a high release probability (failure rate, 23 ± 3%), which is consistent with a monosynaptic connection (Fig. 4*B*,*C*). At a holding potential of −40 mV, the IPSC had a mean amplitude of 20 ± 3 pA, a 10–90% rise time of 1.7 ± 0.1 ms, and a decay time constant of 19.2 ± 1.5 ms. Connections were predominantly unidirectional (*n* = 42 of 45 connected pairs; Fig. 4*B*), with only 3 connected pairs displaying bidirectional connectivity (Fig. 4*C*,*D*). Apart from the stuttering cells, these neurons resembled medium-spiny neurons, (Fig. 5*A–C*), and spine density did not differ significantly between presynaptic and postsynaptic neurons (Fig. 5*B*); nor were differences observed in soma diameter, soma volume, number of primary dendrites, number of nodes, or total dendrite length (Table 2). Recordings were made throughout the rostrocaudal extent of the CeL, and the resulting map of connected and unconnected pairs revealed no obvious location preference (Fig. 5*D*).

**Table 2:** Morphologic properties of neurons in the central lateral amygdala

	Soma length (μm)	Soma volume (μm^3^)	Number of primary dendrites	Number of nodes	Total dendrite length (μm)
Total (*n* = 8)	15.6 ± 0.8	1117 ± 232	5.5 ± 0.4	13.2 ± 1.0	1389 ± 88
Presynaptic (*n* = 4)	14.4 ± 0.9	929 ± 354	5.2 ± 0.6	14.7 ± 0.6	1309 ± 152
Postsynaptic (*n* = 4)	16.9 ± 1.2	1304 ± 322	5.7 ± 0.5	11.7 ± 1.7	1469 ± 93

Values are the mean ± SEM. Four connected pairs (total of eight neurons) were recovered, and their morphologies were analyzed. When these properties were compared between presynaptic and postsynaptic neurons, no significant differences were observed (Mann–Whitney test).

**Figure 6.**
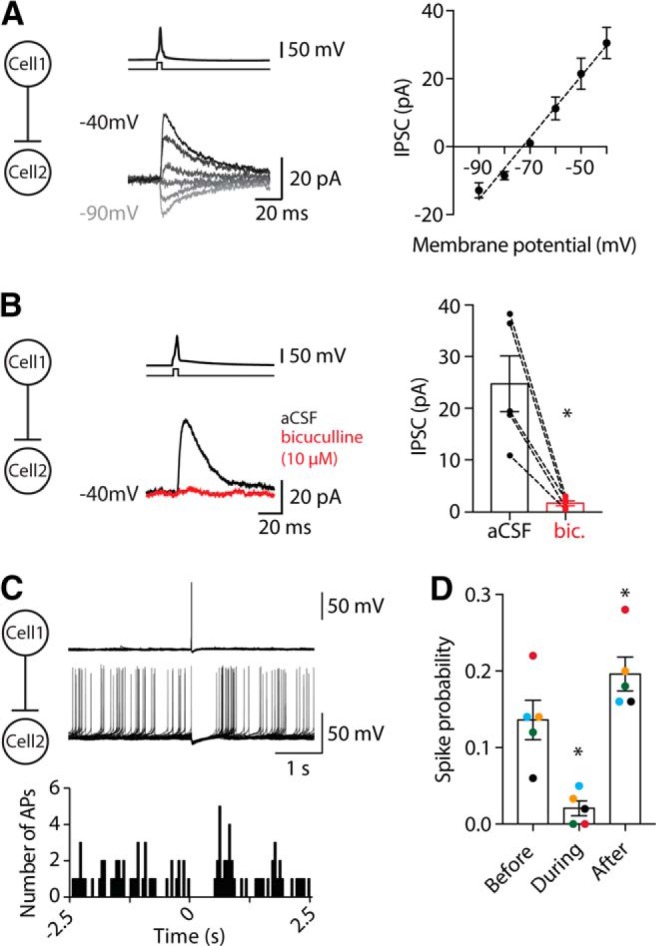
Local connections in the CeL are inhibitory. ***A***, Example traces of change in current to voltage in 10 mV steps (left, from −40 to −90 mV) and average current−voltage (*I–V*) curve of local IPSCs (right, *n* = 5 paired recordings). This *I–V* curve is typical of a chloride current: a linear *I–V* relationship (*r* ^2^ = 0.98) that reverses here at −72 mV, close to the theoretical reversal potential (∼73 mV). ***B***, Local IPSCs were also blocked by the GABA_A_ receptor antagonist bicuculline (10 µM); example traces with aCSF in black and bicuculline in red (left). IPSCs were completely blocked by bicuculline (right, mean IPSC aCSF: 24.7 ± 5.4 pA; mean IPSC bicuculline: 1.7 ± 0.5 pA; *n* = 5 paired recordings; *p* = 0.03, one-tailed Wilcoxon test; dotted line joins data points from the same neuron). ***C***, Overlay of 10 example traces from a connected pair where a short positive current injection (5 ms, 600–700 pA) was applied to the presynaptic cell to fire one AP at *t* = 0 s (top trace). Meanwhile, the postsynaptic cell was also in current-clamp mode, and current was injected such that the cell fired continuously (bottom trace). A single AP in the presynaptic cell evoked an IPSP that was sufficient to stop the postsynaptic cell from firing. Bottom histogram shows the number of APs fired in the above trace over time, in 50 ms bins. ***D***, The spike probability was significantly lower in the 200 ms following inhibition onset compared with preinhibition (mean spike probability before inhibition, 0.14 ± 0.02; mean spike probability during inhibition, 0.02 ± 0.01; *p* = 0.02, paired *t* test), and in most cases increased when the postsynaptic cells recommenced firing (mean spike probability before inhibition, 0.14 ± 0.02; mean spike probability after inhibition, 0.2 ± 0.02; *p* = 0.01, paired *t* test). Each color represents data points from the same neuron (*n* = 5 pairs).

Neurons in the CeL are predominantly GABAergic, and in our connected pairs the IPSC reversal potential was −72 mV, which corresponds to the calculated chloride reversal potential (approximately −73 mV; Fig. 6A). Application of the GABA_A_ receptor (GABA_A_-R) antagonist, bicuculline (10 µM) blocked these IPSCs (Fig. 6*B*; *n* = 5 paired recordings), confirming that they were GABA_A_-R-mediated chloride currents. In current clamp, these connections were hyperpolarizing, with a mean amplitude of −1.1 ± 0.3 mV (*n* = 17), which is sufficient to halt firing in the postsynaptic cell (Fig. 6*C*; *n* = 5 paired recordings), and in some cases this inhibition was followed by a rebound increase in spike probability (Fig. 6*D*). These results demonstrate that neurons throughout the CeL form local inhibitory connections at a relatively high rate, which are capable of shaping the activity of the postsynaptic cell.

### Distinct connection patterns exist between local CeL neurons

**Figure 7.**
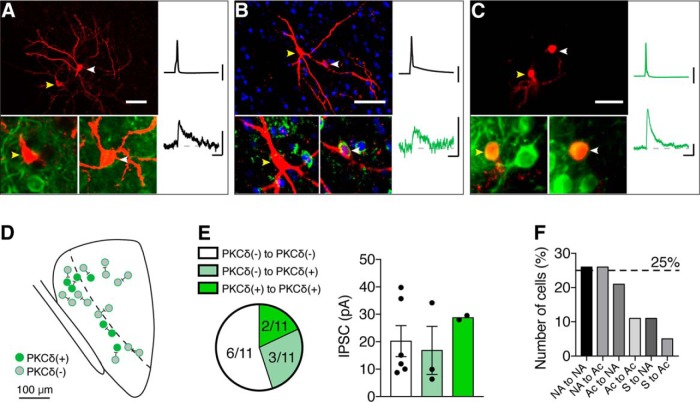
PKCδ(+) and PKCδ(−) neurons form local connections in the CeL. ***A–C***, Example images (left-hand panels; scale bars, 50 µm) of connected cells that were biocytin filled and recovered with a fluorescent streptavidin (red). Insets show closeups of each cell with PKCδ staining (green fluorescence; DAPI is shown in blue in ***B*** to help locate the postsynaptic neuron). Yellow arrowheads indicate the presynaptic neuron, and white arrowheads indicate the postsynaptic neuron. Example average traces for each recovered pair are shown in the right-hand panels, Calibration: 50 mV, 10 pA, 20 ms. ***A***, PKCδ(−) → PKCδ(−) connection. ***B***, PKCδ(−) → PKCδ(+) connection. ***C***, PKCδ(+) → PKCδ(+) connection. ***D***, Approximate locations of each successfully identified pair. ***E***, Connected paired recordings were predominantly between PKCδ(−) cells (∼ 55%; 6 of 11 successfully recovered and stained connected paired recordings), whereas ∼27% of connections were PKCδ(−) → PKCδ(+) (3 of 11) and ∼18% (2 of 11) were PKCδ(+) → PKCδ(+). No PKCδ(+) → PKCδ(−) connections were observed in these experiments. IPSC amplitudes of each type were as follows: PKCδ(−) → PKCδ(−), 20.23 ± 5.6 pA; PKCδ(−) → PKCδ(+), 16.8 ± 8.7 pA; PKCδ(+) → PKCδ(+), 28.75 ± 0.7 pA. ***F***, In terms of firing properties, the majority of connections occurred between LF-NA → LF-NA (∼26%, *n* = 5 of 19), LF-NA → ES-Ac (∼26%, *n* = 5 of 19), and ES-Ac → LF-NA (∼21%, 4 of 19) neurons. ES-Ac → ES-Ac connections were less common (∼11%, 2 of 19), and in all connections that involved a stuttering (S) neuron (∼16%, *n* = 3 of 19), the S neuron was the presynaptic cell.

To determine the identity of recorded pairs, recovered neurons were processed using immunohistochemistry. As expected (Ciocchi et al., 2010; Haubensak et al., 2010), we found local connections between presynaptic PKCδ(−) and postsynaptic PKCδ(+) neurons [PKCδ(−) → PKCδ(+)] in 27% of successfully recovered pairs (Fig. 7*B*,*D*,*E*). However, the most common connection type was between two PKCδ(−) neurons [PKCδ(−) → PKCδ(−)] (∼55%; Fig 7*A*,*D*,*E*). In two cases, both the presynaptic and postsynaptic neurons were PKCδ(+) (18%; Fig 7*C–E*). No PKCδ(+) → PKCδ(−) connections were found. Connected cells displayed a variety of discharge properties (Fig. 7*F*), with the most common connections being either LF-NA → LF-NA (∼26%; *n* = 5 of 19 paired recordings) or ES-Ac → LF-NA connections (∼21%; *n* = 4 of 19 paired recordings). Although less common, we also found ES-Ac → ES-Ac connections (∼10%; *n* = 2 of 19 paired recordings). Stuttering neurons were always presynaptic (*n* = 3), with two connections to LF-NA neurons and one to an ES-Ac neuron.

**Figure 8.**
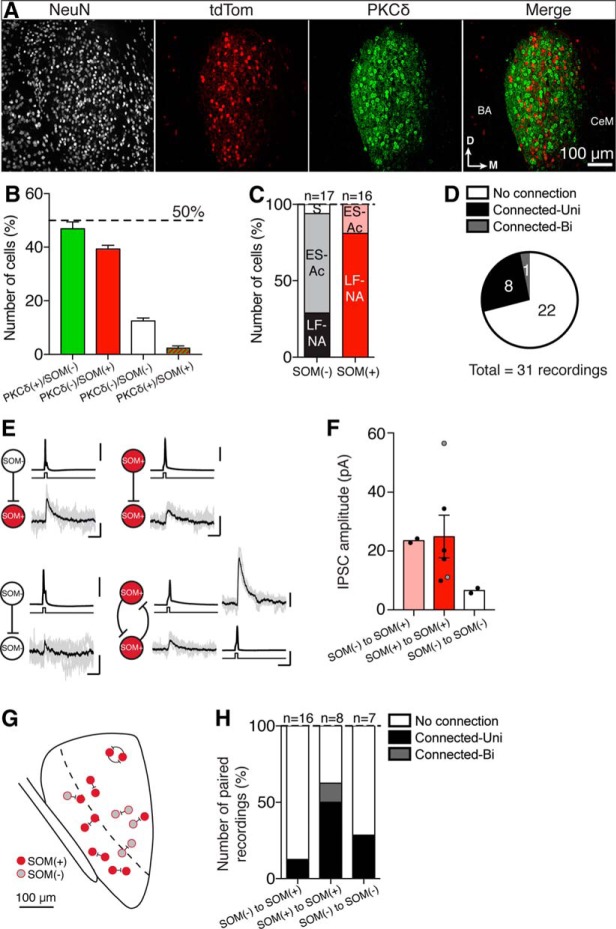
Somatostatin-positive neurons form local connections in the central lateral amygdala of somatostatin-cre mice. The CeL of SOM-Cre C57BL/6J mice was injected with an AAV-DIO-tdtomato to fluorescently label SOM(+) cells. ***A***, Subsections (50 µm thick) of injected CeL were stained with a NeuN antibody and a PKCδ antibody. Representative sections at bregma −1.60 mm are shown. ***B***, NeuN-positive cells were counted for PKCδ and SOM labeling; 47 ± 3% (mean, *n* = 122 ± 27 neurons/1.3 × 10^−3^ mm^3^) of total counted neurons were PKCδ(+) but SOM(−), whereas 39 ± 1% (mean, *n* = 100 ± 16 neurons/1.3 × 10^−3^ mm^3^) of total neurons were SOM(+)/PKCδ(−), with very little overlap [i.e., SOM(+) and PKCδ(+): 2 ± 1% (mean, *n* = 3 ± 1 neurons/1.3 × 10^−3^ mm^3^) and 12 ± 1% negative for both (mean, *n* = 32 ± 6 neurons/1.3 × 10^−3^ mm^3^)]. ***C***, Whole-cell recordings were performed and complete firing properties for 33 neurons were recorded from SOM(+) and SOM(−) neurons. As with wild-type mice LF-NA (∼55%), ES-Ac (∼42%) and stuttering (S; 3%) neurons were observed. SOM(−) neurons were mostly ES-Ac (∼65%, LF-NA 29%, S 6%, *n* = 17 neurons), whereas SOM(+) neurons were mostly LF-NA (∼81%, ES-Ac 19%, *n* = 16 neurons). ***D***, ∼29% of paired recordings showed either a unidirectional (*n* = 8 paired recordings) or bidirectional (*n* = 1 paired recording) connection, whereas in 71% of recordings no connection was detected. ***E***, Unidirectional connections were observed between different combinations of SOM(−) and SOM(+) neurons: SOM(−) → SOM(+) (*n* = 2); SOM(+) → SOM(+) (*n* = 4); SOM(−) → SOM(−) (*n* = 2); and one bidirectional connection was recorded that occurred between two SOM(+) neurons. Calibration: 50 mV, 20 pA, 20 ms. Current injection applied to the presynaptic cell is illustrated in black under each trace. ***F***, Shows IPSC amplitudes for each connection type: SOM(−) → SOM(+) mean amplitude, 23.5 pA (*n* = 2 pairs); SOM(+) → SOM(+) mean amplitude, 24.9 ± 7.3 pA (*n* = 5 pairs – 4 unidirectional IPSCs, 2 bidirectional IPSCs); SOM(−) → SOM(−) mean amplitude, 6.6 pA (*n* = 2 pairs). Gray dots represent IPSCs from the bidirectional connection. ***G***, Diagram showing the approximate location of connected paired recordings within the CeL. ***H***, Shows the number of paired recordings where a connection either was or was not detected for each SOM(+) and SOM(−) combination. A connection was more likely to be observed when recording from two SOM(+) neurons (∼62% connection success rate) as opposed to a SOM(−) → SOM(+) (∼12% connection success rate) or a SOM(−) → SOM(−) combination (∼28% connection success rate).

These results show that local CeL connections occur between a variety of immunohistochemically and electrophysiologically distinct neuronal types with the most common connection between PKCδ(−) neurons. Given that ∼75% of PKCδ(−) neurons are SOM(+) (Fig. 1), we turned to a SOM-Cre mouse line to reliably identify and selectively activate SOM(+) neurons *in vitro*. It was important to confirm that neurons considered to be PKCδ(−) were not false negatives due to protein washout during whole-cell recordings. To label SOM(+) neurons, we injected an adeno-associated virus containing a DIO-td-tomato vector (AAV-DIO-tdTom) into the CeL of SOM-Cre mice (Fig. 8). SOM-tdTom and PKCδ labeling in the CeL revealed proportions of these markers that were similar to those in wild-type mice (Fig. 8*A*,*B*; *n* = 3 mice; at bregma, −1.40 to −1.60 mm). We also determined the firing properties of SOM(+) and SOM(−) neurons (Fig. 8*C*). In agreement with recordings in wild- type mice, SOM(+) neurons were mostly LF-NA (∼81%; *n* = 13 of 16 neurons; ES-Ac: ∼19%; *n* = 3 of 16 neurons), whereas the SOM(−) neurons were mostly ES-Ac (∼65%; *n* = 11 of 17 neurons; LF-NA: ∼29%; *n* = 5 of 17 neurons). Notably, the one stuttering neuron found in these recordings was SOM(−). Given that the stuttering neurons observed in wild-type mice were PKCδ(−), it is possible that these neurons are a major contributor to the population of PKCδ(−)/SOM(−) neurons.

Next, paired whole-cell recordings were obtained using identified SOM(+) neurons (Fig. 8*D–F*). Thirty-one pairs of neurons were recorded, as follows: 8 pairs between SOM(+) neurons; 16 pairs between a SOM(+) neuron and a SOM(−) neuron; and 7 pairs between SOM(−) neurons (Fig. 8*D–G*). Nine of the 31 pairs were connected (∼29%), which included eight unidirectional connections and one bidirectional connection (Fig. 8*D*). In these connections, the mean IPSC amplitude (at −40 mV) was 21 ± 5 pA (*n* = 9) and had an onset latency of 0.76 ± 0.11 ms, which is not significantly different from the results obtained in wild-type mice (wild-type mean IPSC: 20 ± 3 pA; *p* = 0.7, Mann–Whitney test). The IPSC 10–90% rise time was 1.3 ± 0.1 ms and had a decay time constant of 13.2 ± 1.9 ms. The most common connection (∼56%) was between SOM(+) neurons (Fig. 8*E*), with the remaining connections being SOM(−) → SOM(−) (∼22%) and SOM(−) → SOM(+) (∼22%; Fig. 8*E–G*). When we compared the number of connected pairs to the total number of recordings for each combination, the least likely connection was between SOM(+) and SOM(−) neurons, with only ∼12% (*n* = 2 of 16 recordings) of these pairs being connected. In contrast, ∼62% (*n* = 5 of 8 pairs) of SOM(+)/SOM(+) recordings and ∼28% (*n* = 2 of 7 pairs) of SOM(−)/SOM(−) recordings were connected (Fig. 8*H*). No SOM(+) → SOM(−) connections were found.

### Population-driven inhibition is greater between like neurons

#### Somatostatin-positive neurons

**Figure 9.**
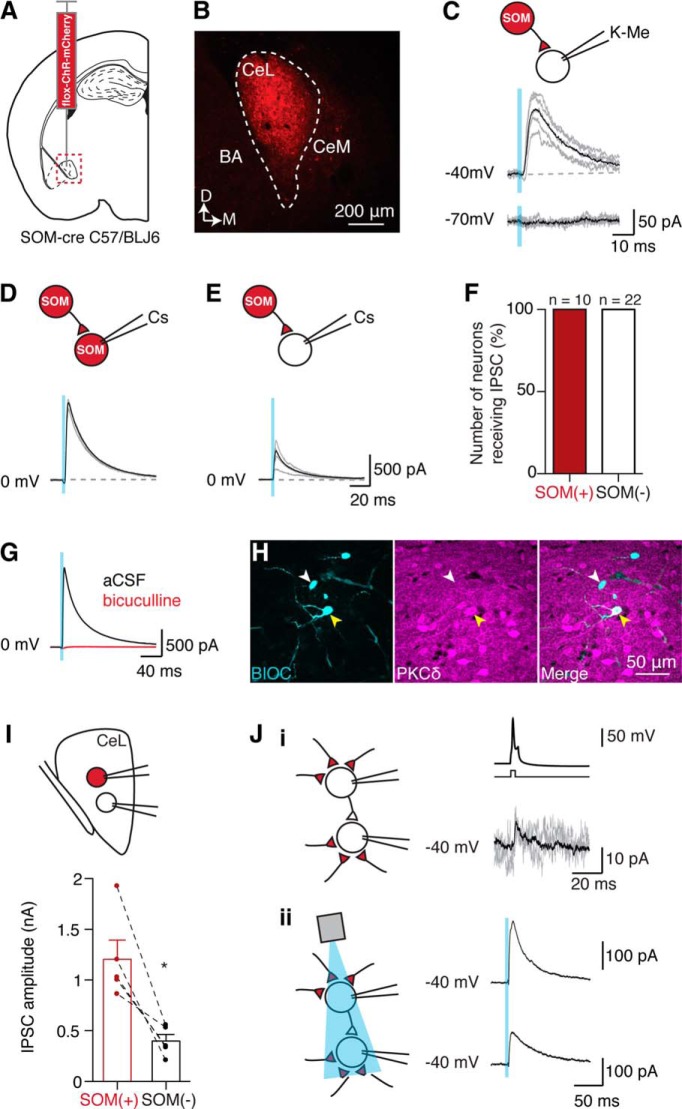
Channelrhodopsin activation of SOM terminals in the central lateral amygdala. ***A***, AAV-DIO-channelrhodopsin-mCherry was injected into the CeL of SOM-Cre C57BL/6J mice. ***B***, Example image of fluorescence of injection site in the CeL (BA, basal amygdala; CeM). ***C***, Using a KMeSO_4_ internal solution (K-Me), we recorded responses from SOM(−) cells in response to a short light pulse (2 ms, 470 nm; blue rectangle; example voltage-clamp traces at −40 and −70 mV), resulting in an IPSC (mean amplitude: 162 ± 24 pA, *n* = 15 cells). ***D–F***, To determine whether all cell types received inhibition from SOM(+) CeL neurons, we also used a cesium-based internal solution (Cs), allowing voltage clamping at 0 mV (ChR reversal potential); average traces are shown in black, and example individual traces are shown in gray. SOM(+) neurons responded with large IPSCs in response to light activation (***D***), as did SOM(−) cells (***E***). ***F***, Light-activated IPSCs were detected in 100% of SOM(+) cells (*n* = 10 neurons) and 100% of SOM(−) cells (*n* = 22). The overall mean amplitude in SOM(+) neurons was 1358 ± 231 pA (*n* = 10 neurons; light pulse: 2 ms, 470 nm), and the mean amplitude in SOM(−) neurons was 609 ± 202 pA (*n* = 12, light pulse 2 ms, 470 ms; the remaining 10 neurons were tested with a 1 ms light pulse: mean amplitude 294 ± 70 pA). ***G***, Bicuculline (10 μM) blocked SOM(+)-driven IPSCs (aCSF mean amplitude, 450 ± 206 pA; bicuculline mean amplitude, 11 ± 4 pA; *p* = 0.04, one-tailed paired *t* test). ***H***, SOM(−) neurons that received SOM(+)-driven inhibition were recovered and stained for PKCδ (*n* = 10 neurons). Five of these neurons were PKCδ(+), while the remainder were PKCδ(−). Example images are shown with biocytin recovery shown in cyan (left), PKCδ staining shown in purple (middle), and the merge shown in the right-hand panel. The white arrowhead indicates one PKCδ(−) neuron, and the yellow arrowhead indicates one PKCδ(+) neuron across all three panels. ***I***, To exclude variation in ChR2 infection and light intensity, and therefore to allow direct comparison of light-evoked IPSC amplitudes, we performed simultaneous recordings from one SOM(+) neuron and one neighboring SOM(−) neuron within the same slice (top diagram). SOM(−) cells typically had smaller IPSCs than their neighboring SOM(+) cell (SOM(+) mean amplitude, 1206 ± 188 pA; SOM(−) mean amplitude, 399 ± 64.8 pA; *p* = 0.01 unpaired *t* test, Welch’s correction; bottom graph, dotted lines join cells that were recorded at the same time, *n* = 5 paired recordings). ***J***, In two cases, light stimulation of SOM(+) terminals during connected paired recordings was possible. ***Ji***, Here, a connected SOM(−) → SOM(−) paired recording is shown with example traces of the connection. ***Jii***, Both the SOM(−) presynaptic and postsynaptic cells of this pair also received SOM(+) inputs. These recordings were conducted using a KMeSO_4_ internal solution.

As described above, paired recordings in coronal brain slices from both wild- type, and SOM-Cre mice show that connections were most frequent between somatostatin expressing PKCδ(−) neurons. However, previous studies indicate that the inhibition of SOM(−) neurons by SOM(+) cells not only exists, but plays a key role in fear expression (Li et al., 2013; Hou et al., 2016). Such a motif is also suggested by inhibition of PKCδ(+) neurons by PKCδ(−) neurons (ON neuron → OFF neuron; Ciocchi et al., 2010; Haubensak et al., 2010). One possibility for our low incidence of SOM(+) → SOM(−) connections is that we are sampling local connections (∼50–100 µm apart) in the coronal plane, and SOM(+) → SOM(−) connections may be more common among “distal” (i.e. >100 µm) connections. To address this, we injected an AAV-containing DIO-channelrhodopsin-mCherry into the CeL of SOM-Cre mice (Fig. 9*A*,*B*) to directly activate SOM(+) terminals.

Whole-cell recordings were made from SOM (+) and SOM(−) neurons, and synapses made by SOM(+) neurons were activated optically. All SOM(−) cells received input from SOM(+) neurons with a mean IPSC of 162 ± 24 pA (*n* = 15; holding voltage, −40 mV; Fig. 9*C*). Next, paired recordings were made using a Cs-based internal solution, allowing voltage clamping of cells at the ChR2 reversal potential (∼0 mV) to test for SOM(+) → SOM(+) connections. In this configuration, all SOM(−) and SOM(+) neurons received large IPSCs when SOM(+) terminals were activated [SOM(−) = 22 neurons; SOM(+) = 10 neurons; Fig. 9*D–F*]. IPSCs in response to SOM(+) terminal activation were fully blocked by bicuculline (10 µm, *n* = 5, Fig. 9*G*), reversed at approximately −67 mV (*n* = 4), and were able to halt firing in the postsynaptic cell. From this cohort, 10 SOM(−) neurons were recovered, of which 5 were PKCδ(+), showing direct SOM(+) → PKCδ(+) and SOM(+) → PKCδ(−) connections (Fig. 9*H*). While all neurons received input from SOM neurons in the CeL, overall input to SOM(+) neurons was significantly larger than to SOM(−) neurons (Fig. 9*I*). This difference is consistent with our paired recordings where five of eight SOM(+) → SOM(+) pairs were connected, but none of the SOM(+)/SOM(−) pairs were (*n* = 16 pairs). In the course of these recordings, it was clear that, using SOM as a neuronal marker, a wide variety of connections are present in the CeL. Thus, for example, in one SOM(−) → SOM(−) single connected pair (illustrated in Fig. 9*J*), both cells also received input from local SOM(+) neurons.

#### Somatostatin-negative neurons

**Figure 10.**
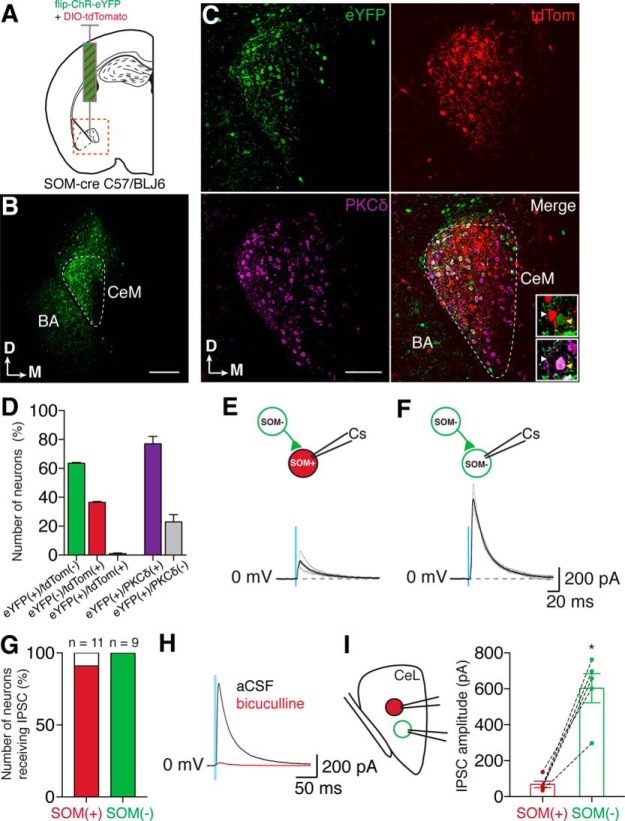
Channelrhodopsin activation of SOM(−) terminals in the CeL of SOM-cre mice. ***A***, to confirm whether SOM(−) neurons in the CeL also form local connections, we injected an AAV-forward-channelrhodopsin-eYFP mixed with an AAV-DIO-tdTomato into the CeL of SOM-cre mice; infected SOM(−) neurons express ChR2-eYFP but not tdTomato (tdTom), whereas SOM(+) neurons express tdTom but not ChR2-EYFP. ***B***, Example image of maximal spread of ChR2-YFP expression at the injection site; the area shown corresponds to the orange square in ***A***. Although the injection covered the majority of the CeL (outlined in white), eYFP(+) somas can still be seen above the CeL and in the BA. Scale bar, 200 µm. Dorsal (D) and medial (M) orientation are shown in the bottom left corner. ***C***, Closeups of the CeL in slices that were also stained for PKCδ. ChR2-eYFP (green), tdTom (red), PKCδ (purple), and merged panels are shown (BA, basal amygdala). Scale bar, 100 µm. Insets in the merged panel show closeups of two neurons from a merged image of eYFP and tdTom stainings (top) and a merged image of eYFP and PKCδ staining (bottom). Arrowheads indicate the same neurons in both insets: a tdTom(+)/eYFP(−) neuron that was PKCδ(−) (white arrowhead), and a tdTom(−)/eYFP(+) neuron that was PKCδ(+) (yellow arrowhead). ***D***, Neurons were counted; 62% were eYFP(+)/tdTom(−) (mean *n* = 67 ± 5 neurons/0.9 × 10^−3^ mm^3^), and 36% were eYFP(−)/tdTom(+) (mean *n* = 39 ± 4 neurons/0.9 × 10^−3^ mm^3^). Theoretically, there should be no overlap of eYFP(+) and tdTom(+) as the presence of Cre recombinase should either allow the expression of tdTom or prevent the expression of ChR2-eYFP. In reality, however, we did observe an overlap between eYFP(+) and SOM(+) neurons, although this was only ∼2% of fluorescently labeled neurons, which represented one to three neurons per 0.9 × 10^−3^ mm^3^ of CeL. The majority of eYFP(+) neurons were also PKCδ(+) (77%; mean, *n* = 51 ± 2 neurons/0.9 × 10^−3^ mm^3^), whereas 23% (mean, *n* = 16 ± 5 neurons/0.9 × 10^−3^ mm^3^) were PKCδ(−). ***E***, ***F***, Whole-cell recordings (CsMeSO_4_ internal solution) of SOM(+) (***E***) and SOM(−) neurons (***F***) revealed that both neuronal types displayed light-activated IPSCs from SOM(−) neurons (SOM(+) mean amplitude: 73.0 ± 19.7 pA; SOM(−) mean amplitude: 427.2 ± 77.8 pA). Example traces are shown with average traces in black and example individual traces in gray. ***G***, Ten of 11 (91%) recorded SOM(+) neurons showed a response to light activation of SOM(−) terminals, whereas 9 of 9 of SOM(−) neurons received inhibitory terminals. ***H***, Bicuculline (10 μm) blocked SOM(−)-driven IPSCs (aCSF mean amplitude, 375 ± 137 pA; bicuculline mean amplitude, 16 ± 7 pA; *p* = 0.03, one-tailed paired *t* test). ***I***, As with our previous experiments, paired recordings between a SOM(+) neuron and a neighboring SOM(−) neuron allowed us to compare IPSC amplitudes from these two cell types (left diagram). These recordings showed that the amplitude of ChR2-driven SOM(−) → SOM(−) IPSCs was significantly greater than that of ChR2-driven SOM(−) → SOM(+) IPSCs (mean SOM(+) amplitude, 68 ± 18 pA; mean SOM(−) amplitude, 603 ± 81 pA; *p* = 0.002 unpaired *t* test, Welch’s correction).

Our paired recordings also showed that SOM(−) → SOM(−) and SOM(−) → SOM(+) local connections, while not frequent, were present (Fig. 8*E*,*F*). However, with the technique we used (Fig. 8) there was a risk that noninfected (and therefore nonfluorescent) SOM(+) neurons could be misidentified as SOM(−). Although the number of SOM(+) neurons in SOM-Cre mice (Fig. 8*A*,*B*) was consistent with that of wild-type mice (Fig. 1), and despite the fact that we made sure to restrict recordings to well within the spread of infection, we used an alternative approach to confirm the existence of these connections. We again used an optogenetic approach to target SOM(−) neurons of the CeL in SOM-Cre mice with an AAV containing a DIO-Fwd-hChR2(H134R)-EYFP construct (Fig. 10*A*). With this construct, the ChR2-EYFP sequence is “cut out” in the presence of Cre recombinase, thereby ensuring that only Cre^−^ [in this case SOM(−)] neurons express ChR2-EYFP. Combining these injections with a DIO-tdTom-containing AAV (1:1 ratio) allowed simultaneous identification of SOM(+) neurons (tdTom fluorescent) and SOM(−) neurons (eYFP fluorescent and ChR2-expressing). We could therefore selectively activate SOM(−) neurons, all while avoiding misidentification of neurons due to lack of fluorescence. These injections typically covered the majority of the width of the CeL (Fig. 10*B*). However, although a small volume of virus (∼100-200 nl) was injected to minimize spread outside the CeL, we did observe eYFP(+) somas in the basal amygdala and the amygdalostriatal area, which is located dorsally to the CeL. Within the CeL, ∼62% of all fluorescently labeled neurons were eYFP(+)/tdTom(−), whereas tdTom(+)/eYFP(−) neurons accounted for ∼36%. Processing slices for PKCδ revealed that the majority of eYFP(+) neurons were PKCδ(+) (∼77%; Fig. 10*C*,*D*).

Using a Cs-based internal solution, whole-cell recordings were obtained from either SOM(+) (Fig. 10*E*) or SOM(−) neurons (Fig. 10*F*). As eYFP(+) neurons were present in the basal amygdala (Fig. 10*B*), we bath applied CNQX (10 μm) during these recordings to ensure that the recorded IPSCs were monosynaptic. Under these conditions, in ∼91% of SOM(+) neurons (10 of 11 neurons) and all SOM(−) neurons (*n* = 9 neurons), stimulation of SOM(−) terminals evoked an IPSC (Fig. 10*G*), and these responses were GABA_A_-R mediated (Fig. 10*H*). Moreover, SOM(−) → SOM(−) IPSCs were significantly larger than SOM(−) → SOM(+) IPSCs (Fig. 10*I*).

Together with our connected paired recordings, these results are consistent with the presence of SOM(−) → SOM(+) and SOM(−) → SOM(−) connections within the CeL. Furthermore, they suggest that, as with SOM(+) neurons, a high proportion of CeL neurons receive inhibitory local connections from SOM(−) neurons, and with inhibition within the population being stronger than that between populations.

## Discussion

The CeA is generally considered to be the main output nucleus of the amygdalar complex and is divided into the lateral and medial sectors. It contains GABAergic neurons that have been divided into several distinct populations using immunohistochemical and electrophysiological markers. These cells form local, as well as long-range connections, and different cell types have been associated with distinct functional roles (McDonald, 1982; Sun and Cassell, 1993; Jolkkonen and Pitkänen, 1998; Ciocchi et al., 2010; Haubensak et al., 2010; Li et al., 2013). Here, using whole-cell paired recordings and optogenetics, we characterized neurons of the CeL and their intrinsic connections. We find that neurons in the CeL are extensively interconnected, with local connections apparent between all types of neurons, but strongest between like neurons. Moreover, we describe a new type of neuron in the CeL with distinct firing properties. These results highlight the complex intrinsic circuits within the CeL and suggest that particular cell groups identified using current methods, rather than mediating specific behaviors, participate in a range of different circuits.

### Local networks in the CeL

Consistent with previous studies, we found that PKCδ and SOM labeled two separate populations of neurons in the CeL (∼48% and ∼38%, respectively), with very little overlap (∼1–2%), that account for 88% of the total cell population. In response to current injection, these neurons show two types of discharge patterns, late firing (LF-NA) and early spiking (ES-Ac), and their overall incidences (∼52% and ∼39% respectively) were comparable to those previously described in the mouse (Haubensak et al., 2010; Hou et al., 2016). While SOM(+) neurons were mostly LF-NA (∼81%) and SOM(−) neurons (largely PKCδ expressing) were more likely to be ES-Ac (∼65%), these electrophysiological properties could not be used to separate the two populations. A smaller number of neurons (∼12%) were PKCδ(−) and SOM(−). These neurons may express CRF or one of the other peptides that are known to be present in CeL neurons (Cassell and Gray, 1989; Haubensak et al., 2010).

A small number of neurons (∼8%), had faster action potentials and a stuttering phenotype, with bursts of high-frequency AP discharge. This type of neuron has not been previously reported in the mouse CeL, although a similar “fast-spiking” neuron has been described in rare cases in the CeL and CeM of the guinea pig and cat (Martina et al., 1999; Dumont et al., 2002). These neurons were PKCδ(−) in wild-type mice, and the one stuttering neuron in SOM-Cre mice was SOM(−), suggesting that they may reflect a distinct PKCδ(−)/SOM(−) population. Although the role of this particular type of neuron is not clear, paired recordings showed that stuttering neurons were always presynaptic, and in cases where we had successful recovery of dendrites they had an aspiny morphology, different from that of the typically recovered CeL neurons. This, together with its fast-spiking properties, suggests the presence in the CeL of a local interneuron-like cell as opposed to the principal-type neurons typically found in the CeL.

Paired recordings demonstrated that neurons in the CeL were connected with an incidence of ∼29%. In these recordings, we find that at the local level (∼50–100 μm in coronal slices), the most common connection was unidirectional and between two PKCδ(−) or two SOM(+) cells. In agreement with a recent report (Hou et al., 2016), connections between other pairs, as well as bidirectional connections were present but were much less prevalent. We did not, however, find cells that showed clear evidence of autapses, which were reported in ∼15% of neurons in the Hou et al. (2016) study. In contrast, when SOM(+) or SOM(−) neurons were transduced with ChR2, we found that nearly all cells received a large input from both cell types. This difference in connectivity indicates that neurons make long-range connections within the CeL, perhaps in the rostrocaudal plane.

For the SOM neurons, using paired recordings, the monosynaptic connection had a mean amplitude of ∼20 pA (at −40 mV), whereas when SOM neurons were transduced with ChR2, the optically driven IPSC had a mean amplitude of ∼160 pA, showing that on average approximately eight SOM(+) neurons innervate each SOM(−) neuron. In paired recordings, the IPSC had rapid rise times, suggesting that these contacts were likely to be somatic, or close to the soma (Delaney and Sah, 2001), which is consistent with the ability of these connections to halt spiking.

### The CeL and behavior

The role of the CeL in cued fear expression is clear: a large body of data supports a model whereby conditioned stimulus-mediated disinhibition of CeM output drives conditioned fear (Ciocchi et al., 2010; Haubensak et al., 2010; Li et al., 2013). However, it remains unclear how the high level of CeL connectivity (both intra-CeL and extra-CeL afferents) can be reconciled with the increasing number of important behaviors in which CeL activity has been implicated. For example, fear expression has also been suggested to require activation of the parabrachial nucleus (PB) input to the CeL (Han et al., 2015; Sato et al., 2015), and yet this PB → CeL circuit has also been implicated in appetite suppression (Carter et al., 2013; Cai et al., 2014). Meanwhile, other CeL circuits have been shown to underlie the switch between innate and conditioned fear (Isosaka et al., 2015), and anxiety generalization (Botta et al., 2015). Last, as well as forming local inhibitory connections (Li et al., 2013), SOM(+) neurons are also projection neurons that target the PAG (Penzo et al., 2014), and this CeA → PAG projection is engaged in mediating defensive behaviors (Tovote et al., 2016). We have shown that these neurons are also highly interconnected both within and between distinct neuronal populations. Our results suggest that within the CeL, neither cytosolic markers (PKCδ and SOM) nor their electrophysiological properties alone can be used to identify cells engaged in particular behavioral roles.

The physiologic role, if any, of SOM and PKCδ are not known; however, they clearly label separate populations of neurons in the CeL. Developmentally, the CeL has a striatal origin (Medina et al., 2011), and SOM and PKCδ, rather than specifying different populations that mediated different functional roles, should be thought of as lineage markers. We suggest that PKCδ-expressing and SOM-expressing neurons form heterogeneous populations of neurons, with different populations contributing to different behavioral outcomes. Understanding the flow of information through the CeA and its outputs, in a behaviorally specific and relevant manner, will be a challenge for future experiments. Similarly, it will be important to take these additional local circuits into account in further investigations of the CeL circuitry, particularly when judging the effects of pharmacological treatments during *in vivo* studies.

## Synthesis

Reviewing Editor: Pablo Castillo, Albert Einstein College of Medicine Decisions are customarily a result of the Reviewing Editor and the peer reviewers coming together and discussing their recommendations until a consensus is reached. When revisions are invited, a fact-based synthesis statement explaining their decision and outlining what is needed to prepare a revision will be listed below. The following reviewer(s) agreed to reveal their identity: Roger Clem, Mario Penzo.

Your manuscript has been evaluated by two reviewers. The following is a synthesis of the online discussion on your study. The reviewers agree on the high quality of the results and overall merit of your findings. They have also raised some criticisms regarding the interpretation of the results that you should address in your revised manuscript. For example, you should acknowledge that the viral transfection efficiency is not necessarily 100%. They understand you should be able to estimate the typical expression efficiency using co-immunolabeling. They also agree you should articulate more clearly the meaning of your findings beyond indicating that the intrinsic circuitry is more complex than previously thought. Below you will find the reviewers' specific comments. It is expected that the revision of your manuscript requires less than 2 months' work.

----------------------------------------------------------------------------

Reviewer #1

The lateral CeA is involved in many types of behavioral regulation. Recent studies suggest that it contains multiple distinct neuronal populations and that these cell types exhibit differential long-range synaptic connectivity. However, the organization of local connections between various cell types remains less clear. Here the authors provide a detailed examination of inhibitory connections among and between somatostatin and PKC-delta expressing populations. The results indicate that more frequent connections exist within than between cell classes. These findings will help establish what types of local computation are supported by CeA neurons by providing a basic wiring diagram of the two major cell classes and demonstrating the function of associated GABAergic connections. The experiments were performed to a high standard, with complementary analyses leaving little room for alternative interpretation. For a somewhat more specialized audience the results will be of high interest. I have just a few main concerns:

1. The most significant, and surprising, finding is that connection probability is highest within PKCdelta or SST+ neurons. This would not necessarily be predicted given the existing model for threat memory expression involving SST--

2. After struggling to understand authors' perspective, I feel like it would be beneficial for them to articulate more clearly why they think that the intrinsic circuitry exhibits more complexity than previously appreciated. Do they mean that there is more heterogeneity of firing phenotypes? If so, why did they not further parse their analysis of connectivity according to these subclasses? Or by complexity are they referring to the fact that there is more local connectivity within different classes than they anticipated?

3. An interesting question to which the authors seem to have alluded is what types of local interactions exist between projection-specific ensembles in the lateral CeA. Do they have any evidence on this topic or can they explain what implications their data might have?

Reviewer #2

The central nucleus of the amygdala, particularly the lateral portion (CeL), has become a center of attention in the study of fear memory following groundbreaking findings in the early 2000s by the groups of Stephen Maren and Joseph LeDoux. However, while several claims have been made regarding the local circuit organization of the CeL, a thorough interrogation of its intrinsic circuits have been missing. The present manuscript by Hunt et al. does much to fill that gap. Following a set of elegant experiments, they reach the important conclusion that connections between like-neurons are typically stronger than those between neurons of different types in CeL. And although, in light of previous publications, it is hard to interpret the overall meaning of these findings, at the very least they raise an important issue about the complexity of central amygdala circuits. This is of vital importance, because it confirms that most models of central amygdala fear circuits are extremely oversimplified and should be revised. Therefore, the contribution of the present manuscript is significant.

In general, the authors have done a good job at addressing the concerns raised during a previous review of their manuscript. And while I am satisfied with most changes, I want to highlight a few points that deserve attention:

1) Towards the end of the 3rd paragraph of the introduction the authors claim that SOM(+) neurons of CeL are likely to be the so-called CeLon neuons of the classic Ciocchi et al., 2010 paper, but that this has not been demonstrated. I disagree. I paper published this summer by Bo Li and colleagues (Yu et al., 2006 - Journal of Neuroscience) shows that SOM(+) neurons of CeL are activated following presentation of the CS+ in conditioned mice. The manuscript should include this important citation.

2) The results presented in Figure 8, 9 and 10 has an important caveat the authors should highlight. Because in these experiments SOM(+) neurons were labeled using a virus approach, it is possible that neurons that are non-fluorescent in these experiments (referred to as SOM(-)) are in fact SOM(+) neurons that were not infected with the virus. Therefore, connectivity rates in these experiments may be slightly compromised because of this. Particularly for SOM(-)-SOM(-) and SOM(+)-SOM(-) connections. It is not surprising to me that they found the single stuttering neuron in Figure 8c to be SOM(-). This could have been a SOM(+) neuron that didn't get infected by the virus. This problem could have been avoided using a Cre-dependent R26 reporter line. And, while I don't think the authors need to repeat these experiments, they should warn the reader that their approach limits the interpretation of the results.
